# Bile and capsid variation coregulate histo-blood group antigen recognition and serum neutralization potency in GII.12 human norovirus

**DOI:** 10.1128/jvi.01338-26

**Published:** 2026-09-25

**Authors:** Michael L. Mallory, Dev K. Patel, Lily M. Parsons, Miranda L. Hubbard, Juyeon Park, Isaac L. Hughes, Paul D. Brewer-Jensen, Mark R. Zweigart, Yaoska I. Reyes, Matthew C. Wolfgang, Lisa C. Lindesmith, Ralph S. Baric

**Affiliations:** 1 Department of Epidemiology, University of North Carolina at Chapel Hill 154805, Chapel Hill, North Carolina, USA; 2 Department of Chemical Engineering, The University of Texas at Austin 12330, Austin, Texas, USA; 3 Department of Molecular Biosciences, The University of Texas at Austin 12330, Austin, Texas, USA; 4 Department of Microbiology and Immunology, University of North Carolina at Chapel Hill 318275, Chapel Hill, North Carolina, USA; 5 Marsico Lung Institute, University of North Carolina at Chapel Hill 735834, Chapel Hill, North Carolina, USA; University of Michigan Medical School, Ann Arbor, Michigan, USA

**Keywords:** norovirus, neutralizing antibodies, blockade antibodies, histo-blood group antigen, ligand affinity, bile

## Abstract

Human norovirus (HuNoV) is a leading cause of acute gastroenteritis, with young children and the elderly bearing the greatest disease burden. GII strains cause the majority of HuNoV outbreaks globally, with host susceptibility influenced by genetic polymorphisms that regulate mucosal expression of histo-blood group antigen (HBGA) binding ligands. HuNoV viral capsid (VP1) diversity in residues adjacent to the conserved HBGA binding pocket selectively stabilizes ligand interactions. Additionally, select HuNoV capsids demonstrate bile-influenced conformational plasticity, further enabling HBGA engagement. Combined, these factors have the potential to enhance the breadth of binding of HuNoV to HBGAs, driving population susceptibility. Here we characterized two GII.12 outbreak strains that co-circulated cotemporaneously and were divergent at five key VP1 sites. Mutational analysis demonstrated amino acids at VP1 positions 373 and 392 coregulate HBGA ligand binding. Serological analysis demonstrated minimal antigenic impact at these positions. Broader GII comparisons demonstrated variable impacts of bile. Bile improved GII.2 and GII.12 binding to HBGA, in contrast to GII.4, GII.3, and GII.6. Further, bile reduced surrogate neutralizing antibody potency for GII.2 and GII.12 but not GII.4 and GII.6 strains. Collectively, these studies suggest that HuNoV bile-dependence is not a general feature of GII norovirus, but instead can be genotype or strain specific. These data inform how select GII HuNoV strains can modulate capsid residues linked to host-ligand and immune recognition, providing potential mechanistic evidence for continual viral circulation.

**IMPORTANCE:**

Human norovirus-host interactions are complex, influenced by viral diversity, host-ligand expression profiles, and the gastrointestinal environment. Periodically, outbreaks of endemic human noroviruses, like GII.12, demonstrate genetic diversity between viral strains of a genotype. Exploring this, two GII.12 outbreak strains were used to model the effects of genetic changes on the viral capsid ligand and antibody binding. Residue changes between the two strains modulated affinity for histo-blood group antigens that could be compensated for by addition of bile. Although bile improved HBGA-binding, it reduced serum and monoclonal antibody neutralization potency to GII.12. These observations demonstrate the balanced impact of bile-mediated viral capsid conformational plasticity in ligand recognition and potential immune evasion for select GII genotypes. Bile had similar effects for GII.2 HuNoV, but did not impact GII.6 or GII.4 HuNoV HBGA-binding and serum-ligand blocking potency, indicating that GII HuNoV strains potentially utilize multiple mechanisms to ensure host breadth and antigenic diversity.

## INTRODUCTION

Annually, human norovirus (HuNoV) causes ~685 million cases of acute gastroenteritis worldwide and is the leading cause of medically attended acute gastroenteritis (MAAGE) in children under 5 years of age (1). HuNoV infections result in an estimated 150,000 adult deaths and 50,000 child deaths per year, with complications arising from dehydration and malnutrition (2–5). In low- and middle-income countries, roughly 14% of gastrointestinal illness is attributable to HuNoV, while in the United States, HuNoV is the most commonly detected pathogen resulting in MAAGE (6, 7). This substantial disease burden warrants large-scale efforts to develop an effective vaccine to protect susceptible populations. However, extensive antigenic diversity and rapid viral evolution have hindered global efforts.

Residing in the Caliciviridae family, HuNoV is a single-stranded, positive-sense RNA virus (~7.5 kb) composed of three open reading frames (ORFs) (8). ORF1 encodes the RNA-dependent RNA polymerase (RdRp) and other nonstructural proteins, while ORF2 and ORF3 encode the major capsid protein (viral protein one, VP1) and the minor capsid protein (VP2), respectively (9). VP1 is divided into the surface-occluded N-terminal shell (S) and surface-exposed C-terminal composed of protruding P1 and P2 subdomains (10). The VP1 P2 subdomain is the most variable domain, encoding the receptor-binding domain and the majority of well-characterized immunodominant neutralizing antibody epitopes (11–17).

HuNoV comprises ≥10 different genogroups and approximately 50 known genotypes (18). GI, GII, GIV, GVIII, and GIX are all known to infect humans, with GI and GII comprising the majority of infections globally (18–21). GII noroviruses, the predominant global genogroup, have robust genetic diversity with >35 identified genotypes (18). In addition to viral diversity, host genetic variation in expression of histo-blood group antigens (HBGAs) influences susceptibility to HuNoV infection and potential spread of virus (22, 23).

The HuNoV P2 subdomain, the most surface-exposed portion of VP1, interacts with the host HBGA ligands via a conserved ligand-binding pocket (11–13). The HBGAs expressed in the mucosa are under genetic regulation by FUT2 and FUT3 (Fucosyltransferase 2 and 3, respectively) (24). A human secretor phenotype is determined by the expression of the H type 1 precursor mediated by the FUT2 gene. Inactivating mutations in FUT2 yield the nonsecretor phenotype, which results in the expression of a restricted set of HBGAs within the mucosal fluids (25, 26). In secretors, the HBGA precursor molecules can further be diversified into H, A, B, or Lewis a and b antigens. Variation in HuNoV VP1 residues surrounding the conserved HBGA-binding pocket allows the virus to compensate for the diversity of HBGA expression patterns within populations and maximize virus infectivity (27, 28).

The majority (50%–60%) of global HuNoV outbreaks are linked to GII.4 variants. These variants are extensively characterized for antigenicity and predominate through rapid and extensive viral capsid evolution, host/herd immune evasion, and immune imprinting (1, 29–34). While GII.4 variants are the primary GII HuNoV target for vaccine-mediated protection, other less common HuNoV GII genotypes, such as GII.2, GII.12, and GII.17, periodically surge in frequency (35–39). Natural genetic variation seen in GII.4, GII.2, and GII.17 within and around the HBGA ligand-binding site shapes the pool of susceptible hosts by mediating ligand and neutralizing antibody binding strength (14, 27, 40, 41). However, the impact of viral capsid mutations surrounding ligand-binding sites in other GII HuNoV genotypes like GII.12 is less well characterized.

GII.12 noroviruses have continuously circulated at low levels for more than 40 years with sporadic seasonal outbreaks (42). During the 2009–2010 winter season, 39/194 (20%) of CDC-reported norovirus outbreaks in the United States were identified as GII.12 (37). These outbreaks typed as a genetically unique GII.12 strain that was similarly detected in Australia and Hungary in 2009 (43). Additionally, a clinical case study of two familial GII.12 norovirus outbreaks that occurred in January 2010 in Ohio demonstrated GII.12 infections in secretor-positive A, B, and O individuals, indicative of broad recognition of HBGAs (44). However, GII.12 virus-like particles of the strain did not substantially bind to synthetic HBGA moieties or HBGAs expressed in human A, B, and O saliva, highlighting the incomplete understanding of GII.12 HuNoV-ligand interactions and other potential host cofactors engagement to confer viral infectivity. Sequence comparison of the isolated outbreak strains with an ancestral GII.12 strain Aichi76, which previously demonstrated type B saliva binding, identified variation at amino acids clustering around the HBGA-binding sites of the P-domain, speculating a link between amino acid variation and ligand recognition (42, 44).

More recently, GII.12 was the second most common infecting GII genotype after GII.4 in a Nicaraguan birth cohort study conducted from June 2017 to December 2019 assessing norovirus acute gastroenteritis infections during the first 18 months of life (45, 46). Several children were found to have durable GII.12 surrogate neutralizing antibody responses with little cross-neutralization to other genotypes, potentially limiting protection to other cocirculating genotypes during the study period. Two additional serological and monoclonal antibody studies further demonstrated that heterologous GII norovirus exposure from the GII.4 component of experimental norovirus vaccines in adults can both weakly boost titers to divergent GII antigens like GII.12 as well as cultivate broadly cross-GII neutralizing antibodies targeting highly conserved, structurally occluded regions (47, 48).

Previous structural analysis of GII.1 and GII.10 demonstrated the impact of bile acids on the viral capsid conformation. Bile elicits conformational changes that preferentially position amino acids for optimal HBGA ligand binding (49). Superposition of GII.1 and GII.12 sequences revealed conservation of GII.1 bile acid-recognizing residues in GII.12, suggesting a potential impact of bile acids on GII.12 HBGA recognition. Supporting the importance of bile in fundamental viral processes, bile/bile acids are either required for replication or stabilization of several norovirus strains (G1.1, GII.3, GII.17, and GII.4) in human intestinal enteroid cells (50, 51). Furthermore, novel amino acid constellations combined with bile have been shown to improve binding of GII.2 to nonsecretor HBGA ligands, potentially expanding the host range of these viruses (28, 40). Collectively, these studies inform potential mechanisms that contribute to long-term global, low-level circulation of GII.12 and potentially other GII viruses.

To examine the impact of GII.12 genetic diversity on ligand, HBGA, and bile, as well as antibody binding, the capsid sequences of two unique GII.12 strains isolated from geographically distant outbreaks were compared (44, 52). Variation predominantly clustered around known conserved HBGA and predicted bile-acid-binding domains. Residues at positions 373 and 392 within the P2 subdomain of VP1 modulated HBGA ligand recognition in distinct patterns regulated by the presence of bile. GII.12 residue variation at these positions exhibited minimal serum neutralization impact in a surrogate neutralization assay. However, the potency of neutralizing responses to all GII.12 residue variants tested was significantly reduced by the presence of bile within the assay, similar to the known bile-regulated GII.2 HuNoV (40). This phenomenon contrasted with other GII noroviruses, GII.6 and GII.4 SY2012, which do not experience significant changes in ligand affinity or neutralizing antibody potency in the presence of bile. These findings demonstrate a complex but potentially common approach that select GII noroviruses employ to persist, evade host neutralizing antibody responses, and periodically surge in outbreaks by varying capsid amino acid constellations and binding bile. Further, analysis of non-GII.4 strains cultivates a broader understanding of viral capsid diversity and its impact on antigenicity and HuNoV host infectivity, which may improve understanding of why the GII.4 genotype remains dominant in global circulation.

## RESULTS

**Fig 1:**
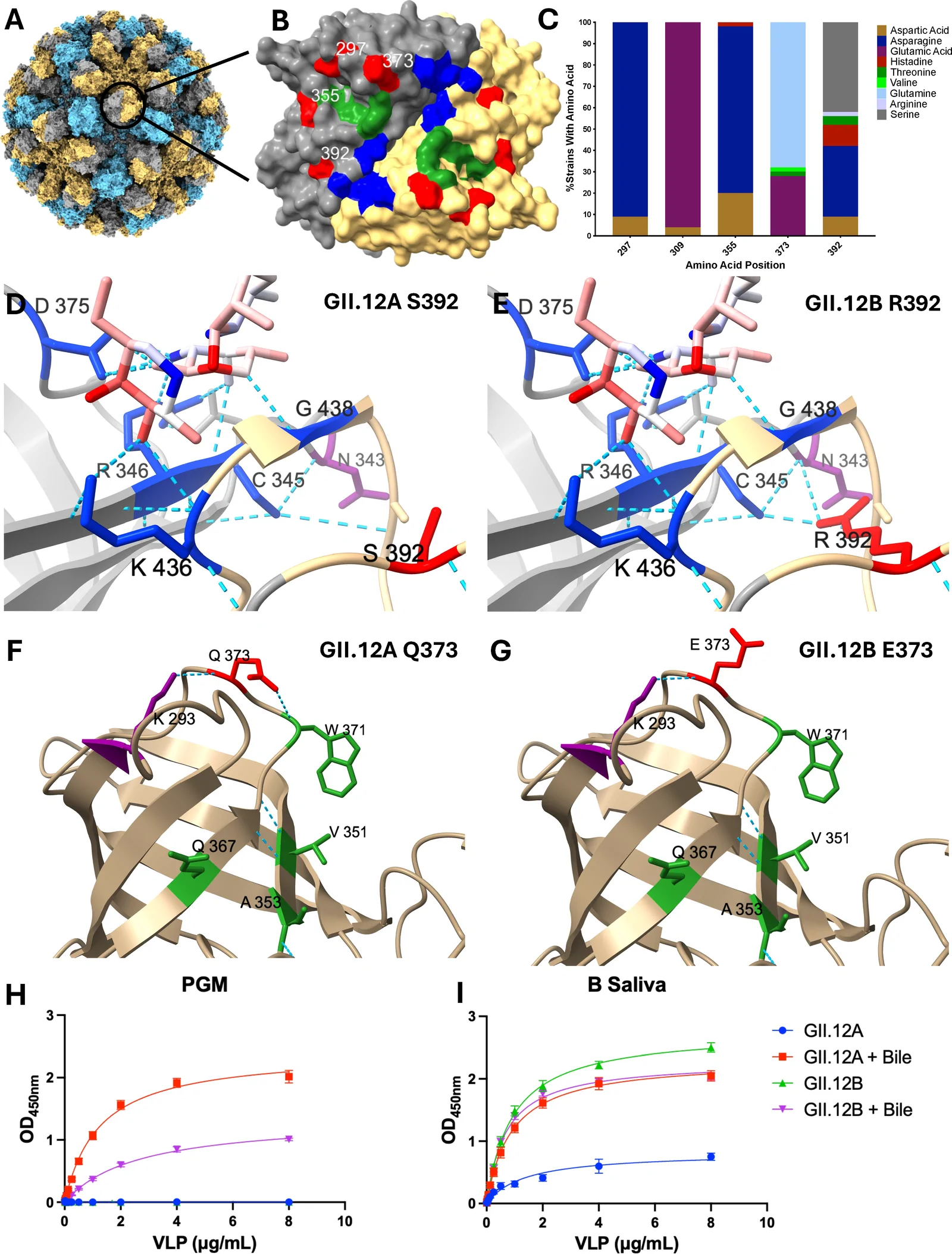
Amino acid variation within VP1 of GII.12 mediates ligand-binding profiles. (**A**) GII norovirus icosahedral capsid structure (GII.4, T = 3, PDB 7YMR), with subunits A, B, and C labeled in dark gray, yellow, and cyan. (**B**) A and B chains (gray and yellow) of the GII.12 P domain dimer (PDB 3R6J) with the five variable residues between GII.12A and GII.12B, labeled in red. HBGA-binding and theoretical bile acid-binding pockets are colored blue and green, respectively. (**C**) Proportional stacked bar graph of the five observed variable sites between GII.12A and GII.12B, with the percentage of amino acid variation at each position calculated from available full-length VP1 GII.12 sequences in GenBank (*n* = 57). (**D–G**) Structural modeling suggests that residues 373 and 392 mediate electrostatic interactions between GII.12 VP1, HBGAs and bile. (**D and E**) GII.12 P domain dimer, dark gray and light yellow, in complex with electrostatically labeled B-trisaccharide, α-l-fucose- (1, 2)-β-d-galactose-(3-1)-α-d-galactose (PDB 3R6K). Residues that directly interact with the B-trisaccharide are labeled in blue. (**F and G**) GII.12 P domain dimer, light yellow, with potential bile-interacting residues labeled in green. Residues 392 and 373 labeled in red, while residues indirectly interacting with either the HBGA or bile acid pocket labeled in purple (**D and E** and **F and G**, respectively). Cyan dashed lines indicate speculative hydrogen bonds formed between P domain residues of interest and B-trisaccharide, when present. Titration of GII.12A and B strains on either porcine gastric mucin (PGM) (**H**) or human B saliva (**I**), with and without 1% bile. Apparent K_d_ and binding maximum (Bmax) for VLP-ligand interactions were determined from the optical density (OD_450nm_) fit to one-site binding curves. Error bars, standard error of the mean (SEM).

To explore the variable HBGA-binding patterns observed among GII.12 strains captured within previous studies, we first examined amino acid diversity between two GII.12 sequences, GII.12A (2010 USA, AEI29586.1) and GII.12B (2010 France, AJP13623.1). Collected during a January 2010 norovirus outbreak in Ohio, GII.12A demonstrated weak HBGA ligand interaction, differing from previously published reports on GII.12B and other GII.12 strains (47, 53, 54). Alignment of these sequences revealed 99.7% amino acid similarity with five amino acid differences found at positions 297, 309, 355, 373, and 392. Mapping these differences on the GII.12 capsid P domain dimer (Fig. 1A and B) revealed four out of five amino acid differences, 297, 355, 373, and 392 clustered near the known conserved GII HBGA B-trisaccharide (α-l-fucose- (1, 2)-β-d-galactose- (3-1)-α-d-galactose) and speculative GII conserved bile acid binding pockets. Observing these differences, we then aligned 57 GII.12 full-length capsid sequences available by 1 March 2026, that emerged over a > 40-year circulation window and analyzed the percent variation that occurred at the five variable sites observed between GII.12A and GII.12B. Positions 392 and 373 were found to have the most amino acid diversity, with six and four different amino acid variants at each location, respectively (Fig. 1C). Further, of the six detected amino acid variants at position 392, five were observed in multiple outbreaks, suggestive of increased immune pressure and toleration of hypervariation at this position.

Noting the increased variation at amino acid positions 392 and 373, closer predictive structural modeling of the GII.12 capsid revealed two interesting patterns. The GII.12 P dimer positions the type B trisaccharide-interacting residues near the interface of the two adjoining capsid monomers. The GII conserved residues C345 and R346 on P-dimer subunit chain A have the potential to indirectly interact with residue 392 on chain B. Compared to the serine present on the GII.12A strain (Fig. 1D), which has a shorter, polar, and uncharged hydroxymethyl side chain, the arginine present on GII.12B has a longer carbon aliphatic side chain with a positively charged guanidinium group (Fig. 1E). The combination of length and basic charge on the GII.12B R392 on chain B is enough to form an additional hydrogen bond with N343 on chain A, which has the potential to structurally stabilize the loop of amino acids functionally interacting with the B trisaccharide as well as support the preferential conformation for GII.12B P-dimer relative to the ligand.

For residue 373, predicted electrostatic interactions are isolated onto a single P-dimer chain. Notably, both amino acid variants at position 373 form a hydrogen bond with a non-bile acid-pocket residue, lysine at 293. Glutamine at 373, Q373, on GII.12A (Fig. 1F and G) is able to form an additional single hydrogen bond with a conserved hydrophobic bile-pocket residue, tryptophan, W371, while E373 in GII.12B does not form this additional contact point. For the GII.12A strain with Q373, this potentially influences stability of the loop of amino acids forming the bile acid-binding pocket, potentially positioning GII.12 hydrophobic capsid residues to better interact with host bile acids.

To examine the impact of amino acid variation between the GII.12 strains, virus-like particles (VLPs) of GII.12A and GII.12B were synthesized (Fig. S1A and B). GII.12A and GII.12B were equally reactive to rabbit antisera to GII.12B VLP (Fig. S2A) and screened for binding to Porcine Gastric Mucin Type III (PGM), a commercially available source of mucin composed of H, A, and Lewis Y antigens, and human type B saliva. Binding interactions were conducted in the presence or absence of 1% bovine bile for maximal bile-related impact (Fig. 1H and I; Fig. S2B and C), with apparent K_d_ and binding maximum (B_max_) values calculated via one-site-specific nonlinear regression analysis (40). Notably, both GII.12 strains required the presence of bile for binding to PGM, with GII.12A demonstrating higher B_max_ (2.42 OD_450nm_ vs 1.36 OD_450nm_, *P* = 0.0002) and improved ligand affinity (lower K_d_ value: 1.24 μg/mL vs 2.60 μg/mL, *P* = 0.0019) to PGM compared to GII.12B. While both GII.12 strains were capable of binding to type B saliva without bile, the GII.12B strain displayed stronger binding affinity (K_d_: 0.30 μg/mL) and B_max_ (2.77 OD_450nm_), compared to GII.12A (K_d_: 1.24 μg/mL and 0.82 OD_450nm_, *P* = 0.0085 and *P* = 0.0159, respectively). For type B saliva binding in the presence of bile, the GII.12B strain did not show a substantial change in ligand-binding affinity (K_d_ without bile 0.30 μg/mL, K_d_ with bile 0.25 μg/mL, *P* = 0.4857), while the GII.12A strain demonstrated a modest improvement in ligand-binding affinity (K_d_: 1.29 μg/mL without bile, 0.89 μg/mL with bile, *P* = 0.3543) as well as a significant increase in ligand binding signal (B_max_, 0.82 vs 2.31 OD_450nm_, *P* = 0.0016).

**Fig 2:**
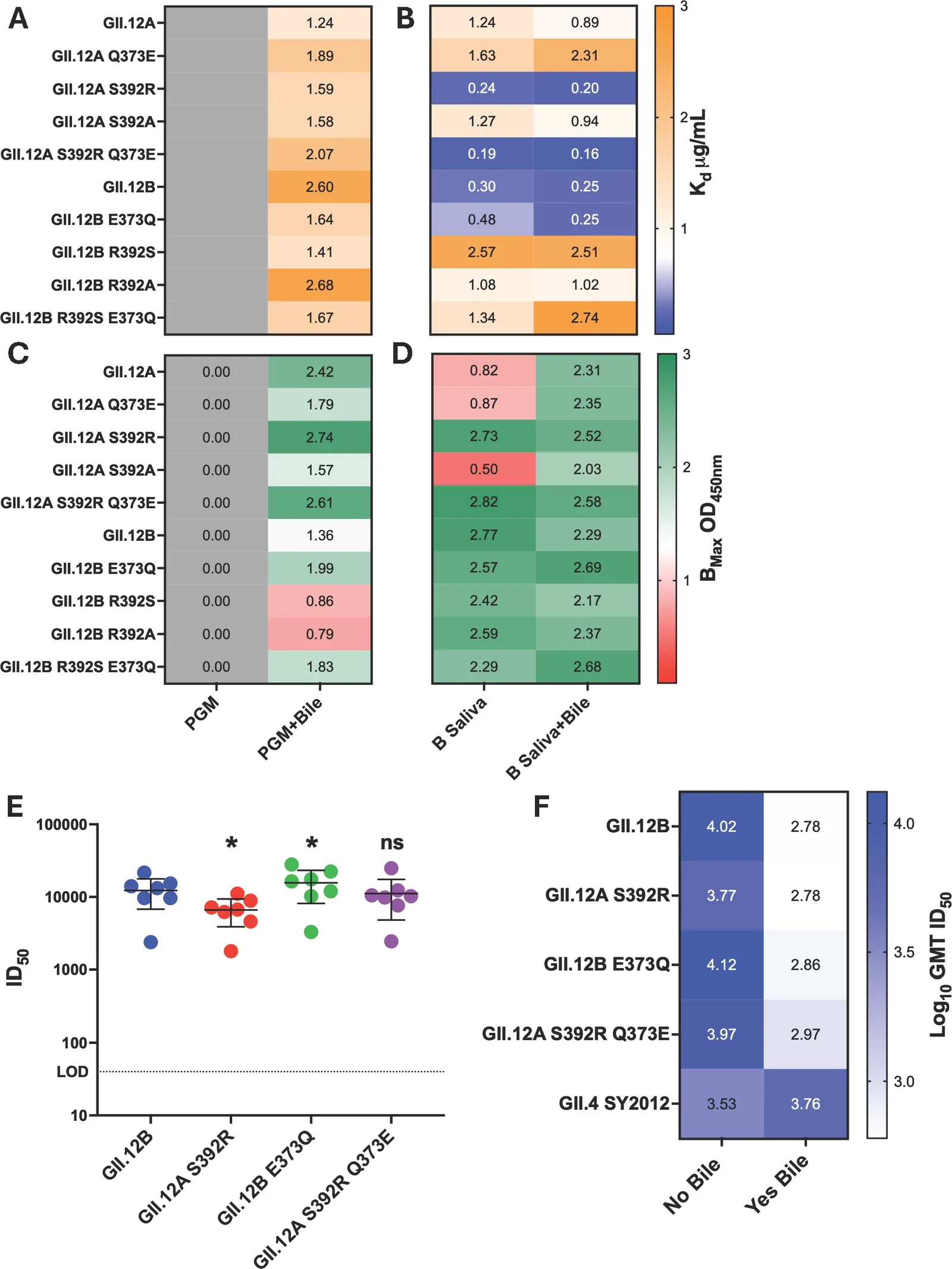
Bile compensates for GII.12 sequence variation in HBGA binding while reducing the potency of serum-neutralizing antibody response. Apparent K_d_ calculated from the ligand-binding titration of GII.12 VLPs with amino acid swaps between GII.12A and GII.12B strains at positions 392 and/or 373, or alanine substitution at position 392, against either PGM (**A**) or human B saliva (**B**) in the absence or presence of 1% bile. Mean optical density (OD_450nm_) was fit using a one-site-specific nonlinear regression analysis, reporting K_d_ and B_max_. Heatmap: blue = lower K_d_ (higher affinity) orange = higher K_d_ (lower affinity), gray = no reported affinity or binding below the assay limit of detection (LOD). (**C and D**) Heatmaps of B_max_ OD_450nm_ values calculated from VLP-binding titration on either PGM or B saliva, with or without bile; red = lowest OD, green = highest. (**E**) Blockade ID_50_ values of GII.12 VLP-ligand binding to human B saliva by GII.12B hyperimmune BALB/c mouse serum (*n* = 7) calculated by nonlinear regression analysis (**F**) Heatmap of serum log-transformed GMT ID_50_ for blockade of VLP-ligand binding to human B saliva by hyperimmune mouse serum to either GII.12B or GII.4 SY2012 in the presence or absence of bile. *, statistically significant, *P* < 0.05 via Wilcoxon paired *t*-test. ns, non-significant.

To elucidate the impact of variation at positions 392 and 373 on the GII.12 capsid, amino acid residue exchanges were introduced using PCR single-site mutagenesis at either single or dual positions between the GII.12A and GII.12B strains. VLPs were synthesized (Fig. S1C through J) with reciprocal 392 exchanges (GII.12A S392R and GII.12B R392S), alanine (392A) substitutions, 373 exchanges (GII.12A Q373E and GII.12B E373Q), or both positions exchanged (GII.12A S392R Q373E and GII.12B R392S E373Q). Titration of all GII.12 capsid mutants on either PGM or human B saliva (Fig. 2; Fig. S3) demonstrated variable binding kinetics to ligands based on the residue at 392 and 373.

**Fig 3:**
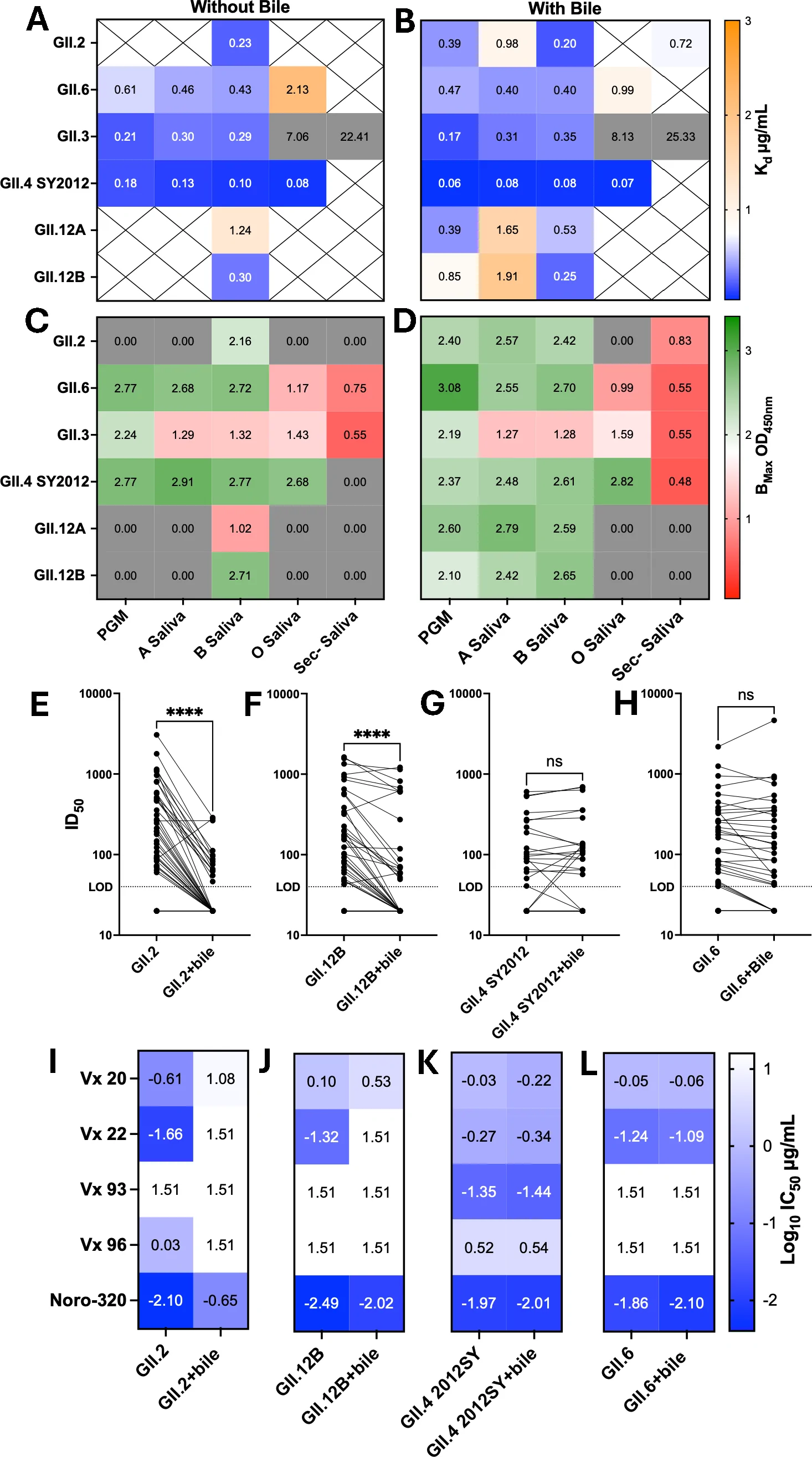
Bile mediates HBGA binding and modulates serum-neutralizing antibody potency of select GII genotypes. A diverse panel of GII noroviruses were titrated against PGM, secretor-positive A, B, and O, and secretor-negative (Sec−) saliva samples with and without 1% bile. Optical density (OD_450nm_) was fit using one-site-specific binding curves with apparent K_d_, representing the relative affinity for the HBGA ligand to the VLP target, calculated. (**A, B**) Heatmaps of K_d_ values transition from blue to orange, representing high to low affinity, respectively, while gray = VLPs that bind to ligand but demonstrate K_d_ values outside an acceptable window of affinity. X = VLPs that do not have detectable binding at any tested concentration. (**C, D**) Heatmaps of B_max_ OD values calculated from VLP titration on HBGA sources, with or without bile; red = lowest OD, green = highest. Serum blockade of ligand-binding antibody response from adult human donors (*n* = 42) was analyzed against GII.2 (**E**), GII.12B (**F**), GII.4 SY2012 (**G**), and GII.6 (**H**) VLPs, in the presence and absence of 1% bile on B saliva. ID_50_ values were calculated by nonlinear regression analysis for each serum sample and compared for each VLP-ligand interaction with and without bile. *, **, ***, ****, and ns are *P* < 0.05, <0.01, <0.001, <0.0001, and non-significant via Wilcoxon-matched pairs sign rank test, respectively. (**I–L**) Cross-GII neutralizing human monoclonal antibodies demonstrating blockade of GII.2, GII.12B, GII.4 SY2012 and GII.6 VLP-ligand binding in the presence and absence of 0.1% bile. IC_50_ values were calculated via nonlinear regression, low to high IC_50_ values, blue to white.

As with the GII.12A and GII.12B strains, all 392/373 mutants required the presence of bile to bind to PGM, with modest variation in ligand affinity (K_d_; Fig. 2A; Table S1). On PGM with bile, GII.12A Q373E and GII.12A S392R Q373E displayed a slight reduction in binding affinity (K_d_: 1.89 and 2.07 μg/mL, *P* = 0.0451 and *P* = 0.0080 respectively), compared to the GII.12A wild-type strain (K_d_: 1.24 μg/mL). The GII.12B E373Q and GII.12B R392S E373Q VLPs demonstrated a slight improvement in binding affinity (K_d_: 1.64 and 1.67 μg/mL, *P* = 0.0009 and *P* = 0.0379, respectively) in comparison to the GII.12B wild-type strain, 2.60 μg/mL, (Table S2) for statistical comparisons. Alanine and arginine at 392 for GII.12A demonstrated a weak but insignificant reduction in K_d_ (1.58 and 1.59 μg/mL, *P* = 0.3511 and *P* = 0.2222) compared to the wild-type GII.12A strain, while alanine at 392 in the GII.12B strain did not influence K_d_ (2.68 μg/mL).

B_max_ data with bile on PGM demonstrated a similar impact of residue changes at 373 and 392 (Fig. 2C). In the GII.12B residue swap VLP, the E373Q residue exchange in either the single or 373–392 double mutant improved B_max_ (1.99 and 1.83 OD_450nm_, *P* = 0.0206 and *P* = 0.0002 respectively) compared to the parental GII.12B (1.36 OD_450nm_). Additionally, GII.12B R392S and R392A single mutants reduced B_max_ (0.86 and 0.79 OD_450nm_, *P* = 0.0016 and P = 0.0006) compared to the parental GII.12B strain. For the GII.12A residue swap VLP, the Q373E and S392A single mutants significantly reduced B_max_ signal (1.79 and 1.57 OD_450nm_, *P* = 0.0062 and *P* = 0.0025) compared to the parental GII.12A strain (2.42 OD_450nm_), while the S392R and 373-392 double mutant (2.74 and 2.61 OD_450nm_, *P* = 0.1274 and *P* = 0.3450) did not substantially improve the B_max_ signal.

Screening the GII.12 capsid mutants on type B saliva revealed more specific binding patterns in comparison to PGM. All GII.12 VLPs bound to B saliva in the absence of bile (Fig. 2B; Table S1). All VLPs that had a native S392 residue (GII.12A and GII.12A Q373E, K_d_: 1.24 and 1.63 μg/mL, *P* = 0.0095 and *P* = 0.0095 respectively), or R392S (GII.12B R392S and GII.12B R392S E373Q, K_d_: 2.57 and 1.34 μg/mL, *P* = 0.0159 and *P* = 0.0095), or 392A (GII.12A S392A and GII.12B R392A, K_d_: 1.27 and 1.08 μg/mL *P* = 0.0015 and *P* = 0.0061, respectively) demonstrated lower binding affinity compared to GII.12B containing arginine at 392, K_d_: 0.30 μg/mL (Fig. 2B; Table S2). Further, GII.12A S392R (GII.12A S392R and GII.12A S392R Q373E, K_d_: 0.24 μg/mL and 0.19 μg/mL, *P* = 0.0043 and *P* = 0.0022) as well as GII.12B E373Q (GII.12B E373Q, K_d_: 0.48 μg/mL, *P* = 0.0010) mutants displayed either substantial improvements compared to GII.12A (K_d_ 1.24 μg/mL) or similar B saliva recognition (*P* = 0.4127, *P* = 0.1123, *P* = 0.0539) compared to GII.12B (K_d_: 0.30 μg/mL), pointing to a strong influence of residue 392 on B ligand binding K_d_.

Interestingly, this pattern of B saliva affinity was largely retained in the presence of bile with a weak secondary influence of residue variation at 373. GII.12 VLP containing Q373 (GII.12A, GII.12B E373Q, and GII.12A S392A, K_d_: 0.89, 0.94 and 0.25 μg/mL) all demonstrated weak improvements in ligand binding with bile when compared to their binding without bile (1.24, 1.27, and 0.48 μg/mL, *P* = 0.3543, *P* < 0.0001, and *P* > 0.999). GII.12B VLPs containing E373 (GII.12B R392S and GII.12B R392A, K_d_: 2.51 and 1.02 μg/mL) demonstrated similar binding affinity to type B saliva with bile compared to without bile (2.57 and 1.08 μg/mL, *P* > 0.9999 and *P* = 0.8048). GII.12A VLP containing Q373E demonstrated weak but insignificant improvements to K_d_ (2.31 vs 1.63 μg/mL, *P* = 0.2468). Similar to PGM binding, maximum binding of all VLPs was either retained for VLPs that recognize type B saliva well or substantially improved for low-affinity-binding VLPs such as GII.12A, GII.12A Q373E, and GII.12A S392A, B_max_ OD_450nm_ 2.31, 2.35, and 2.05 with bile vs 0.82, 0.87, and 0.50 OD_450nm_ without bile (*P* = 0.0016, *P* = 0.0043, and *P* < 0.0010) (Fig. 2D; Table S2).

Observing these ligand-binding differences, a subset of GII.12 single-site amino acid mutants were analyzed against increasing concentrations of primary bile acids GCDCA, TCA, or bovine bile to further understand the interaction between bile and GII.12 capsid variation at positions 373 and 392 (Fig. S4). On PGM, increased concentrations of bile acids and bile demonstrated reduced ligand recognition of the GII.12A Q373E mutant (Fig. S4A through C), with B_max_ comparisons for GII.12A Q373E vs GII.12A of GCDCA (0.17 vs 0.39 OD_450nm_), TCA (0.14 vs 0.33 OD_450nm_), and bile (0.47 vs 0.78 OD_450nm_). A similar reduction in PGM binding was observed with the GII.12B R392S mutant compared to the GII.12B and GII.12B E373Q VLPs (Fig. S4D through F), with B_max_ comparisons for GII.12B R392S vs GII.12B of GCDCA (0.08 vs 0.27 OD_450nm_), TCA (0.09 vs 0.27 OD_450nm_), and bile (0.35 vs 0.66 OD_450nm_). On B saliva, binding patterns were similar in the presence of either bile acid and bile, but more heavily influenced by the presence of R392 (Fig. S4G through L). Collectively, these data suggest a coregulation of PGM and type B saliva binding with GII.12 residues 392 and 373 in the absence and presence of bile and primary bile acids.

Next, to understand the impact of amino acid variation between the GII.12A and B capsids on antigenicity, hyperimmune sera raised against GII.12B in HuNoV-naïve mice were tested against the GII.12 VLPs that bound well to type B saliva in the absence of bile in a surrogate neutralization assay based on antibody blockade of VLP-ligand binding. Compared to GII.12B, the ID_50_ GMTs to the tested GII.12 capsid variant VLPs were within ±2-fold (1.78×, 0.79×, and 1.12× for GII.12A S392R, GII.12B E373Q, and GII.12A S392R Q373E, respectively) (Fig. 2E). While the titer differed significantly for GII.12A S392R and GII.12B E373Q VLPs compared to GII.12B, these <2-fold differences in GMT observed between the tested GII.12 VLPs indicate that 392 and 373 are not key variant-specific antibody-binding residues.

In the presence of bile or individual bile acids, conformational changes can occur on the viral capsid that improve the affinity for GII.12 (and other GII noroviruses like GII.2) binding to HBGA ligands (40). To elucidate the potential impact of bile on mouse sera antibody recognition and surrogate neutralization potency within the GII.12 VLP panel, serum blockade assays were repeated on B saliva with the addition of 1% bile (Fig. 2F). For each GII.12 VLP, the ID_50_ GMT in the presence of bile was substantially lower compared to the ID_50_ GMT of assays conducted in the absence of bile: GII.12B (17-fold decrease), GII.12A S392R (10-fold decrease), GII.12B E373Q (18-fold decrease), and GII.12A S392R-Q373E (10-fold decrease), respectively. Juxtaposed to GII.12, mouse serum blockade titer to a divergent GII, GII.4 SY2012, was modestly increased in the presence of bile (1.7-fold) (49). Collectively, these data suggest that while bile does not differentiate amino acid variation between GII.12A and B, it overall has the potential to induce conformational changes on the GII.12 capsid that improves affinity for some HBGAs and significantly reduce serum blockade potency.

Altered ligand-binding properties have been attributed to GII.4 norovirus persistence (16, 27). To evaluate if the identified impact of bile and residue variation in GII.12 strains could also potentially impact host susceptibility patterns, VLP-binding assays were conducted against a panel of secretor-positive human salivary samples (*n* = 20). Secretor-positive salivary binding trends for GII.12 VLPs with bile were similar to B saliva and PGM binding patterns (Fig. S5; Table S3). In the absence of bile, GII.12 VLPs containing R392 generally demonstrated stronger and broader salivary binding than S392 or A392 VLPs. Across the saliva panel, 1% bile greatly increased binding for all GII.12 mutants, with VLPs containing Q373 generally demonstrating a larger improvement in binding signal than those containing E373.

To explore if bile had an impact on ligand binding or neutralizing antibody potency, representative VLPs for GII.2 (QLI46383.1), GII.3 (AFK75854.1), GII.6 (CRL46967.1), GII.4 (AGJ52172.1), and GII.12 were titrated in ligand-binding assays against secretor-positive A, B, O, and secretor-negative human saliva in the absence and presence of bile (Fig. S6). Without bile present, all tested genotypes bound to type B saliva (Fig. 3A and C; Table S4). While GII.2 and GII.12 strains were limited in binding only to type B saliva, GII.3, GII.4 SY2012, and GII.6 also bound to PGM, type A, and type O saliva. GII.3 and GII.6 each also exhibited weak binding to secretor-negative saliva. Ligand binding of GII.3, GII.4 SY2012, and GII.6 (Fig. 3B and D) was not bile-dependent. However, bile improved binding for GII.2 and GII.12 to PGM and type A saliva (Fig. 3B and D). As previously reported, bile improved binding affinity of GII.2 to only secretor-negative saliva (40).

Noting the variable impact of bile on GII capsid-ligand interactions, GII.2, GII.12B, GII.6, and GII.4 SY2012 VLPs were assessed for serum blockade of ligand-binding titer on type B saliva with a panel of commercially available human serum collected prior to 2021 in the absence and presence of bile (Fig. 3E through H). Representing population sera with unknown exposure history, individual serum titers were variable for all four VLPs. In the presence of bile, serum GMT blockade titers were substantially reduced for GII.2 and GII.12B (5.58×-fold and 2.37×-fold, respectively), while GII.4 SY2012 and GII.6 remained largely unchanged (1.03×-fold and 1.20×-fold).

Finally, the impact of bile on select GII norovirus capsid-ligand interactions was assessed for surrogate neutralization potency against a panel of neutralizing cross-GII human monoclonal antibodies (HumAb) (Fig. 3I through L) (48, 55, 56). Previous structural analysis of these cross-GII HumAbs demonstrates epitope binding within conserved and structurally occluded regions on the HuNoV capsid that are conformationally regulated. For GII.4 SY2012 and GII.6, bile demonstrated minimal impact on surrogate neutralization potency of VX20, VX22, VX93, VX96, and Noro-320, with an IC_50_ ±1.6×-fold change. Against GII.2, two mAbs (VX22 and VX96) experienced a complete loss of potency in the presence of bile at the highest HumAb concentration tested, while VX20 and Noro-320 experienced 50.0×- and 27.8×-fold reduction in neutralization potency. Like GII.2, bile ablated VX22 neutralization potency for GII.12B, while moderately impacting neutralization potency for VX20 and Noro-320 (2.7×-fold and 3.0×-fold). Notably, non-neutralizing HumAbs in this panel did not improve potency in the presence of bile. While these HumAbs target unique structurally occluded and conserved regions on the GII HuNoV capsid distal to the HBGA and bile-binding pockets, they provide additional supporting evidence of how bile can induce conformational changes more broadly across the viral capsid to potentially limit cross-protective HuNoV immunity. Collectively, these studies illustrate how varying capsid amino acid constellations can impact complex interactions between the virion, cell entry ligands, and neutralizing antibodies in select GII HuNoV.

## DISCUSSION

The extensive genetic diversity of HuNoV poses a complex challenge for understanding how these viruses interact with genetically diverse hosts and evade cross-protective immune responses to facilitate global circulation. Prevalent GII.4 variants are characterized by antigenic drift in neutralizing antibody epitopes, RdRp/capsid gene recombination, broad HBGA ligand affinity, and cocirculating episodic strains (14, 31, 57–59). However, for less common GII HuNoVs like GII.12, mechanisms for sustained low-level circulation are not as well understood. Understanding how genetic variation within the VP1 capsid protein for non-dominant genotypes like GII.12 is critical for understanding how these viruses continue to circulate within human populations.

Here we employed VLPs of the VP1 capsid protein to compare the ligand binding and antigenic differences between two geographically isolated GII.12 strains circulating within the United States (GII.12A) and France (GII.12B) in 2010 (44, 52). Noninfectious VLPs, which are structurally similar to native virions, demonstrate conformational plasticity influenced by temperature and host cofactors, including bile (49, 60, 61). These external factors have the potential to regulate both antibody and ligand recognition of VLPs and virus by mediating particle conformational dynamics.

Sequence variation that clustered around the conserved HBGA-binding and hydrophobic bile acid-binding pockets at positions 392 and 373 of GII.12 demonstrated the highest degree of amino acid variation among 57 GII.12 capsids in GenBank. VLP mutational analysis between GII.12A and B strains at positions 392 and 373 demonstrated that these residues modulate ligand-binding affinity to HBGA ligands. These changes were influenced by the presence of bile, which broadened the HBGA-binding profile to mimic the breadth of bile-independent GII noroviruses like GII.3, GII.4 SY2012, and GII.6. This was further exemplified through the analysis of HBGA secretor-positive human donor saliva binding of GII.12 VLPs, demonstrating how bile-induced capsid interactions can compensate for natural HuNoV genetic variation within host HBGA ligand binding. While microvariation on the GII.12 capsid did not impact polyclonal serum reactivity, the surrogate neutralizing antibody potency for some serum and monoclonal antibodies decreased for GII.12 and GII.2 in the presence of bile. In contrast, bile had little impact on serum and monoclonal neutralizing potency against GII.4 SY2012 and GII.6 VLPs, collectively illustrating a unique balance of HuNoV genotypic complexity between capsids, binding cofactors, and neutralizing antibodies.

Cultivating a deeper understanding of how HuNoV viral capsid diversity influences binding of host cofactors like bile is essential for understanding the dynamics behind virus-host interactions. Recent GII.2 HuNoV VLP-based studies revealed that within genotype strains, diversity improved HBGA-binding breadth in the presence of bile (40). Additionally, studies utilizing protein X-ray crystallography have demonstrated the selective binding certain genotypes of HuNoV like GII.1, GII.10, and GII.19 display to primary bile acids, in comparison to non-bile-recognizing genotypes like GI.1, GII.3, GII.4, and GII.17 (49). Structural analysis demonstrated residue conservation within the defined bile acid pocket on the capsid protein was able to stabilize P domain loops adjacent to the carbohydrate-binding sites to either enable (GII.1) or enhance HBGA binding (GII.10) in the presence of bile acids (49). Collectively, these studies support our findings, as capsid microvariation at residues adjacent to the conserved HBGA- and bile-binding pockets of GII.12 differentially modulated HBGA pocket interactions.

Supporting these HuNoV structural characterization studies and ligand-based assays, live-virus studies have shown the importance of bile for replication of select HuNoV genotypes (GI.1, GII.3, GII.17, and GII.4) and porcine enteric calicivirus within the Human Intestinal Enteroid and porcine cell culture systems (62–64). Further, mouse norovirus has been shown to experience a complex balance of increased CD300lF receptor engagement in the presence of bile while simultaneously occluding previously defined monoclonal antibody-binding sites (65–67). In addition to altering the structure of individual particles, bile and hydrophobic bile acids have the potential to promote aggregation of HuNoV VLPs, creating high-avidity clusters that influence attachment to HBGA and entry into human intestinal enteroid cultures (68). As vaccine-mediated protection from severe HuNoV illness is prioritized, these findings inform on the importance of further studies assessing the unique equilibrium between antigenic diversity, epitope presentation, host-gut cofactors like bile, and humoral immunity.

Compared to GII.4s, there is a dearth of resources available to assess the impact of capsid genetic variation on infectivity or antigenicity for non-GII.4 strains. Surveillance systems, which are essential for capturing real-time norovirus circulation, often publish only partial capsid sequences. While fragmented VP1 sequencing supports important epidemiological questions, it limits the capacity to track and characterize unique capsid amino acid patterns. To date, out of the 317 GII.12 VP1 sequences in GenBank, only 57 report >90% of VP1. Additionally, limited live-viral GII.12 isolates that are unique and characterized within the HIE system hinder the ability to understand VP1 genetic variation within infection-based studies. Modeling off of GII.4 VLP-based mutational studies, which have a larger pool of full-length VP1 sequences to characterize host-ligand and antibody response within strains, here we explored the two most variable sites documented on GII.12 (27) through ligand binding and blockade of VLP-ligand binding assays. These assays, while surrogate in nature, inform ligand interactions of HuNoV capsids. While the K_d_ and B_max_ analysis here is limited to the biochemical structure of our assay, it can provide future direction to affinity-based studies within the live-viral HIE system that is currently limited by cultivation challenges such as strain diversity, reproducibility, and cost. Additionally, blockade of VLP-ligand assays has previously demonstrated a strong correlation with the limited pool of serum and antibody samples tested within the HIE neutralization assay system. Though our study demonstrated modulated ligand interactions from HuNoV capsid changes, further studies exploring other evolving amino acid positions adjacent to conserved ligand-binding sites like those on GII.12, GII.12 X-ray crystallography, as well as live-viral studies within the HIE system could additionally inform how select GII HuNoV coregulate host-ligand interactions.

Common polymorphisms in the CYP7A1 gene, which initiates bile acid synthesis, and the BAAT gene, which encodes the enzyme crucial for the final synthesis of bile acids in the liver, can up- or down-regulate overall bile expression in humans (69, 70). The inter-individual and inter-population variability of CYP7A1 and BAAT gene expression has the potential to limit the generalizability of bile acid-based HuNoV ligand-interactions. Additionally, bile acid synthesis and composition vary by age. Previously, infants ≤2 years old demonstrated higher proportions of serum-detectable primary bile acids, which decrease and balance with the metabolic conversion into secondary bile acids with increasing age (71, 72). These findings, in addition to waning maternal immunity, immune immaturity, and nutrition, may contribute to increased detection of endemic GII HuNoVs in infant-cohort studies (46).

To address the variability of bile acid genetic and age-based expression, here we focused on a commercially available unfractionated bovine bile upon confirmation of the impact of individual bile acids GCDCA and TCA on GII.12 ligand binding. Compared to individual bile acids, unfractionated bile, which provided the largest increase in GII.12 binding signal to HBGA ligands, consists of a pool of bile acid salts, cholesterol, phospholipids, and proteins, which supplies additional factors found in the gut where HuNoV interacts with both antibodies and cellular receptors. This unfractionated bile provides a more representative example of the gut cofactors in addition to individual bile acids present at the time of infection, enabling HuNoV-HBGA ligand interactions.

Norovirus circulation is omnipresent within the global population, as it persists via several mechanisms, such as genotypic and subgenotypic variation, genetic recombination events, modulation of antibody as well as host cofactor-binding sites, immune imprinting, and waning of cross-reactive responses. By expanding the research focus beyond the globally dominant genotype GII.4, we may discern virologic and immunologic features that distinguish between HuNoV epidemic and endemic events and develop an understanding of how and why endemic genotypes utilize host cofactors to aid in infection, spread, and immune evasion. Further, diversifying our knowledge of endemic genotypes will inform how to design broad immunogens that elicit robust cross-protective immune responses geared at decreasing the global burden of norovirus disease.

## MATERIALS AND METHODS

### GII.12 homology model and sequence alignments

GII.12 P domain dimer (PDB 3R6J), GII.12 P domain dimer in complex with HBGA type B triglycan (3R6K), and GII.4 VLP (7MRY) were modeled in ChimeraX, with electrostatic interactions predicted and selected based on highest conformational likelihood via the rotamers tool (UCSF, Version 1.9) (73, 74). GII.12 VP1 amino acid sequences obtained on 1 March 2026 were aligned using Geneious Prime (Version 2025.2.2) with default parameters.

### Production of virus-like particles

Virus-like particles were produced as previously reported, with VP1 genes optimized for human expression (BioBasic Inc., Markham, ON, Canada) and directly ligated into a pVR21 Venezuelan Equine Encephalitis (VEE) replicon vector using ApaI and AscI restriction sites (16, 60, 75, 76). Sequence verification was performed using Oxford Nanopore long-range sequencing (Plasmidsaurus, San Francisco, CA, USA). Purified pDNA was linearized with NotI (NEB, Ipswich, MA, USA, #R0189S) and transcribed into 5’-capped mRNA using the mMESSAGE mMachine T7 transcription kit (Invitrogen, Waltham, MA, USA, #AM1344). mRNA was then electroporated into BHK-21 cells (ATCC CCL-10, Manassas, VA, USA) for virus-like particle (VLP) production and incubated for 24–27 h at 37°C and 5% CO_2_. VLPs were harvested via ultracentrifugation as previously reported (30, 60). VLP protein concentration was determined using the BCA Protein Assay (Pierce/Thermo-Fisher, Waltham, MA, USA, #23227), and particle integrity was verified via negative-stain electron microscopy.

### PCR point mutagenesis

Targeted VP1 mutations in GII.12A and GII.12B VLPs were produced using the New England Biolabs Q5 Site-Directed Mutagenesis Kit (NEB, Ipswich, MA, USA, #E0554S), according to the manufacturer’s protocol. Next, pDNA was transformed into *Escherichia coli* Top10 cells (Invitrogen, Waltham, MA, USA) and purified using a QIAprep Miniprep Kit (Qiagen, Germantown, MD, USA, #27104). VLPs were produced from sequenced-verified pDNA as described above.

### VLP titration on ligand assay

VLP binding to porcine gastric mucin (PGM) type III (Sigma) and salivary samples was done as previously described (40). Corning (3366) plates were coated with either PGM (10 μg/mL) or de-identified human saliva from secretor-positive type A, B, O, or secretor-negative donors that were heat-inactivated at 100°C for 10 min and clarified via centrifugation at 21,000 × *g* (14,000 rpm) for 5 min and diluted in DPBS for 4 h and blocked overnight at 4°C in 5% non-fat dried milk in PBS-0.05% Tween-20 (Sigma-Aldrich, St. Louis, MO, USA, #P3563). Starting at 8 μg/mL, VLPs were serially diluted 1:2 with or without 1% by weight bovine bile (Sigma-Aldrich, St. Louis, MO, USA, #B3883) on nonbinding dilution plates (ThermoFisher, Waltham, MA, USA, #442587). The VLP-bile mixtures were then transferred to PGM- or saliva-coated plates. Plates were incubated for 1 h at 37°C. Plates were then washed and incubated with GII.2, GII.3, GII.4, GII.6, or GII.12 hyperimmune rabbit sera (custom, CoCalico Biologicals), followed by anti-rabbit IgG-HRP (Cytiva, Marlborough, MA, USA #NA934). Color development was completed using 1-Step Ultra TMB ELISA HRP substrate solution (ThermoFisher, Waltham, MA, USA, #34029) and stopped with 2 M H_2_SO_4_. Plates were read at OD_450nm_. Maximum binding and apparent K_d_ values were determined by a one-site-specific curve fit analysis in GraphPad Prism (10.6.1).

### Bile acid and bile titration on ligand assay

Bile acid and complete bile titration assays were conducted as similarly described. In a 1 μg/mL VLP and 5% non-fat dried milk in PBS-0.05% Tween-20 solution, either GCDCA (sodium glycochenodeoxycholate, Sigma-Aldrich, #G0759) or TCA (sodium taurocholic, Sigma-Aldrich, #T4009) were titrated 3-fold at a starting concentration of 81 mM on nonbinding dilution plates. Bovine bile was similarly titrated, starting at a 3% solution. Plates were then transferred to PGM- or B saliva-coated plates and incubated for 1 h at 37°C. Plates were then washed, and bound VLPs were detected with GII.12 hyperimmune rabbit sera, and color was developed as described above. Plates were read at OD_450nm_, and binding curves fit via one-site-specific curve analysis in GraphPad Prism (10.6.1).

### Saliva binding assay

De-identified human saliva samples were collected under a protocol reviewed and approved by the University of North Carolina at Chapel Hill Institutional Review Board (IRB #20-0792). Salivary samples were heat-inactivated and clarified as described above. Corning (3366) plates were coated with heat-inactivated saliva samples as above, diluted in DPBS for 4 h, and blocked overnight at 4 °C. Plates were then washed and incubated for 30 min at 37 °C with 1.25 μg/mL horseradish peroxidase (HRP)-conjugated UEA-I (Sigma-Aldrich, St. Louis, MO, #L8146). Color development was completed using 1-Step Ultra TMB ELISA HRP substrate solution. Plates were read at OD_450nm_. Samples were phenotyped as secretor-positive or -negative based on UEA-1 reactivity that was 3-fold above PBS-only background. Secretor-positive saliva samples were diluted 1:500 in DPBS and added to duplicate wells of 96-well plates. Saliva-coated plates were incubated at room temperature for 4 h, blocked overnight at 4°C, before the addition of VLPs (1 μg/mL) diluted with or without 1% bovine bile. Binding was detected with GII.12 hyperimmune rabbit sera, followed by anti-rabbit IgG-HRP, and color developed as described above.

### Serum and monoclonal samples

Eight-week-old BALB/c mice were obtained from Inotiv (Strain 047). Mice were submandibularly bled prior to vaccination to establish baseline serum reactivity. Mice were then divided into either GII.12B or GII.4 2012SY vaccine groups, *n* ≥ 5, and intramuscularly vaccinated three times at 4-week intervals with 2 µg of VLP diluted in PBS supplemented with 2% Alhydrogel. Bleeds were taken 3 days prior to each vaccination and 4 weeks after the second boost. Clarified sera were stored at −80°C for long-term storage or 4°C for short-term storage. Commercially available de-identified human sera collected prior to 2020 (BioIVT, Woodbury, NY, USA) from 42 individuals were heat-inactivated at 56°C for 30 min, spun for 2 min at 10,000 × *g*, aliquoted, and stored at −80°C for long-term storage or 4°C for short-term storage. Monoclonal antibodies were a generous contribution from Dr. Juyeon Park and Dr. George Georgiou at The University of Texas at Austin and were isolated as previously described (48, 56).

### Blockade of VLP ligand-binding assay

Surrogate neutralization serum blockade of VLP-ligand binding assays and monoclonal antibody blockade of ligand-binding assays were completed by pretreating VLPs with serial dilutions of serum or concentrations of monoclonal antibody with or without 0.1%–1% bovine bile, as previously described (27, 40, 77). Treated VLPs were then incubated for 1 h at 37°C and subsequently transferred onto ligand-coated plates (either PGM or human B saliva), as described above. VLP-ligand plates were then processed as described above for ligand-binding assays. The percentage of VLP binding in the presence of serum or monoclonal antibody was compared to a no-antibody control, with percent-control binding data analyzed via nonlinear log(inhibitor) vs normalized response-variable slope curve fit in GraphPad Prism and the 50% inhibitory dilution (ID_50_) or inhibitory concentration (IC_50_) calculated. Samples that did not block at least 50% of VLP binding to ligand were assigned an ID50 value of 0.5 times the lowest serum dilution tested or two times the highest µg/mL monoclonal antibody concentration tested for statistical analysis.

### Statistical analysis

Statistical analysis of apparent K_d_ and binding maximum values obtained from one-site-specific curve fit analysis on GraphPad Prism was completed by taking the calculated K_d_ for each technical run and analyzing by group via Mann-Whitney test in GraphPad Prism. Statistical analysis of HGBA-VLP ligand blockade of binding assay results was completed by taking the calculated ID_50_ for each sample determined via nonlinear log(inhibitor) vs normalized response-variable slope curve fit and analyzed by group via Wilcoxon matched-pairs signed-rank test via GraphPad Prism.

## Data Availability

Accession numbers for viral capsid sequences used to develop virus-like particles are available throughout the text. Sequences for human monoclonal antibodies utilized here are publicly available in the cited publications. Additionally, the published protein structure used for predictive modeling here is available in Materials and Methods.

## SUPPLEMENTAL MATERIAL

Supplemental material jvi.01338-26-s0001.pdf Fig. S1 to S6; Tables S1 to S4.

ASM does not own the copyrights to Supplemental Material that may be linked to, or accessed through, an article. The authors have granted ASM a non-exclusive, world-wide license to publish the Supplemental Material files. Please contact the corresponding author directly for reuse.
