# Supplementary material for: Bile and capsid variation coregulate histo-blood group antigen recognition and serum neutralization potency in GII.12 human norovirus
Source: J Virol. 2026 Sep 25;100(10):e01338-26. doi: 10.1128/jvi.01338-26 (PMC13712811; doi:10.1128/jvi.01338-26)
Supplement: Supplementary file 1 [file jvi.01338-26-s0001.pdf]

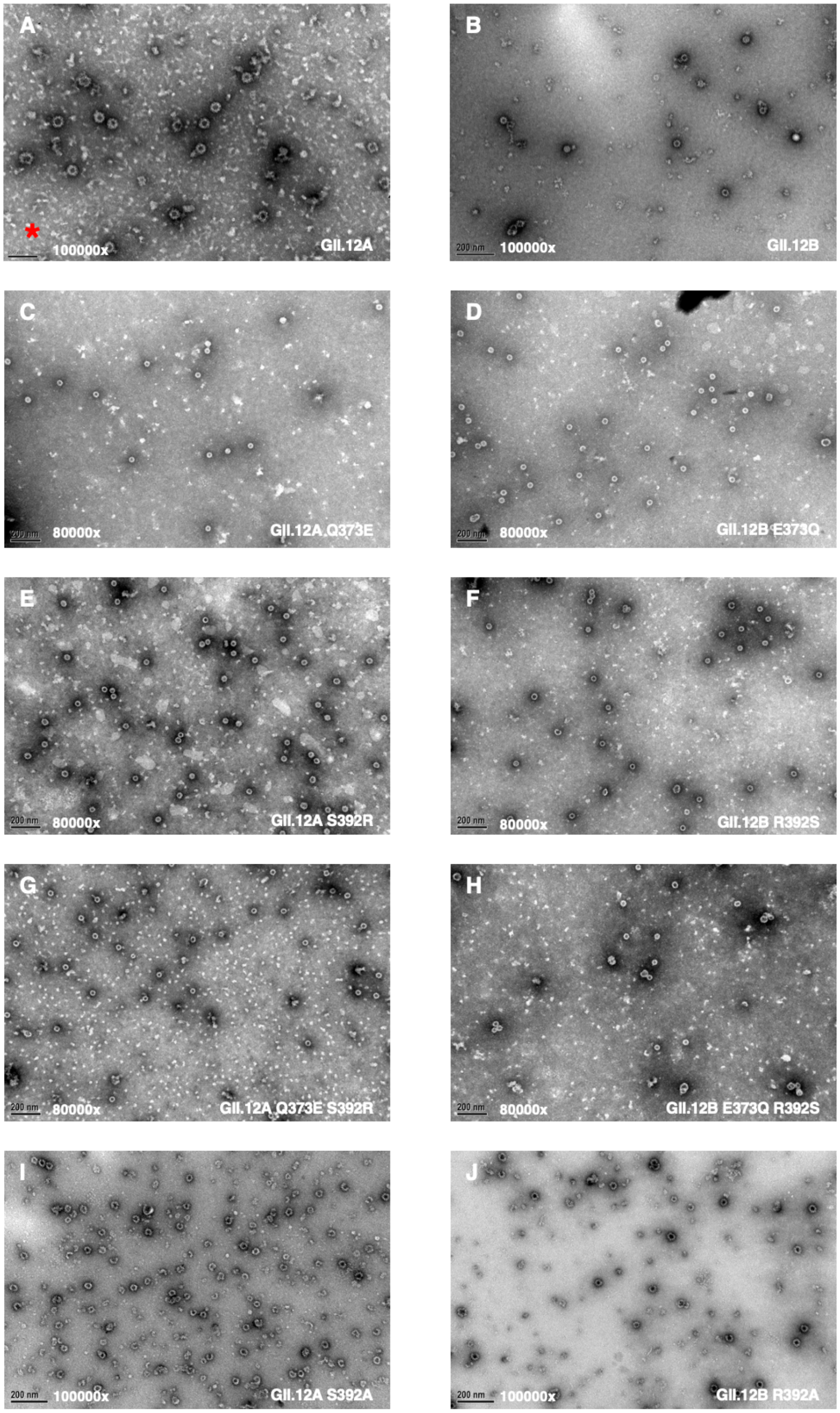

Figure S1: Negative stain electron micrographs of GII.12 virus like particles. Red asterisk on panel A, scale = 100nm.

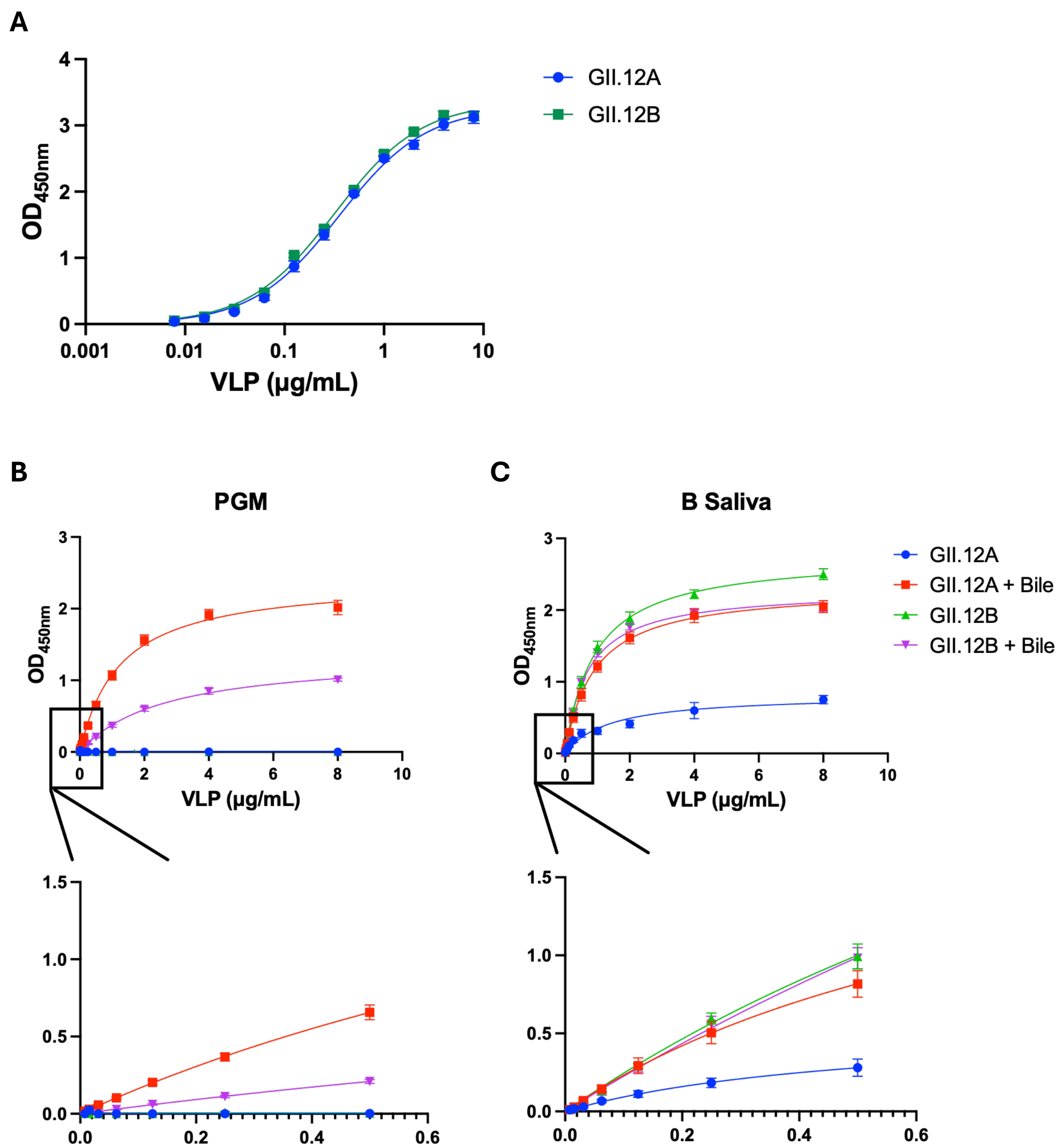

Figure S2: GII.12 serological and ligand binding detection. (A) Detection of GII.12 VLP binding to ligand detected by rabbit antisera to GII.12B VLP. Titration of GII.12A and B strains on either PGM (B) or human B Saliva (C), with corresponding graphical insets focusing on VLP concentration range of 0.008-0.5μg/mL. VLP titrations conducted with or without 1% bile. OD were fit by one-site specific non-linear regression curve fit analysis, error bars representing standard error of the mean.

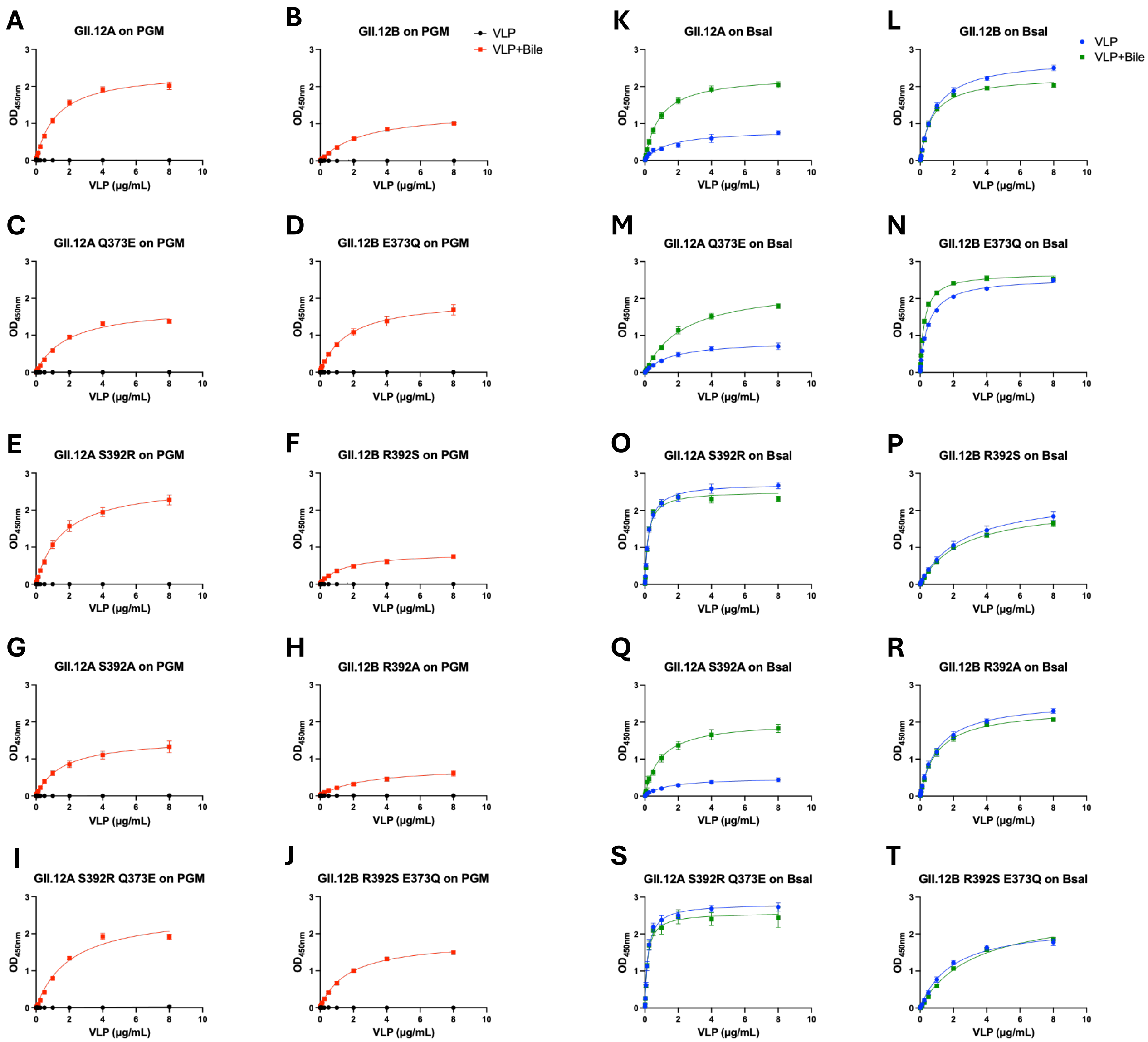

Figure S3: Bile and residues 392 and 373 cooperatively influence GII.12 ligand binding. GII.12A and GII.12B VLP with residue swaps at positions 392 and 373, or alanine substitution at position 392 were titrated for binding to PGM (A-J) or human secretor positive type B saliva, (K-T) in the absence or presence of 1% bile. GII.12A, and VLP utilizing the GII.12A backbone, red=no bile and black=bile (A,C,E,G, I and K,M,O,Q,S), or GII.12B backbone, blue = no bile and green = bile (B,D,F,H,J and L,N,P,R,T). Data were analyzed using one-site specific non-linear regression curve fit analysis with error bars representing the standard error of the mean.

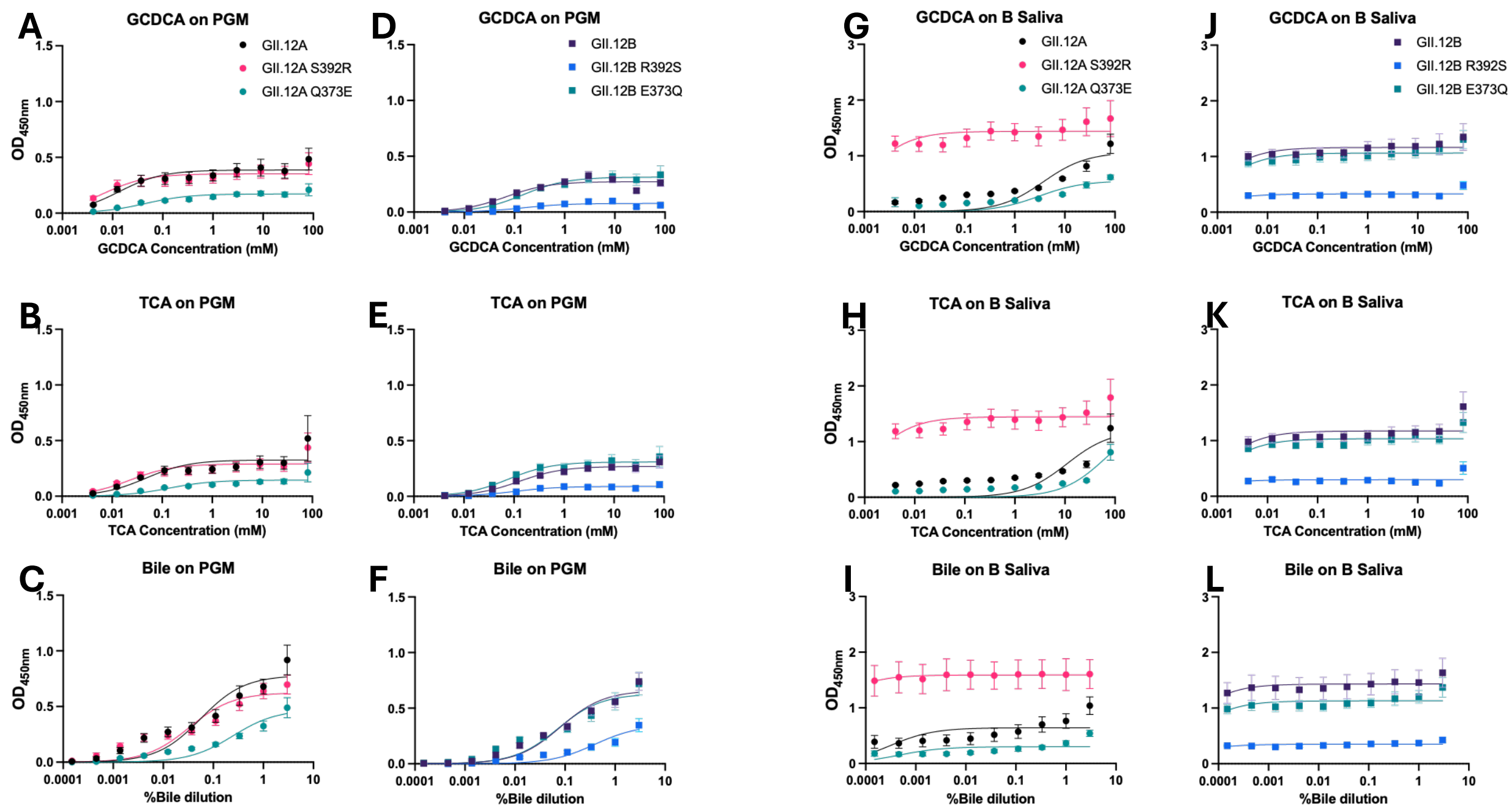

Figure S4: Primary bile acids GCDCA, TCA and complete bile facilitate binding of GII.12 VLP to ligands. Optical density binding at 450nm of GII.12A (A-C) and GII.12B (D-F) VLP to PGM, and B saliva (G-L) in the presence of increasing concentrations of primary bile acids GCDCA (A, D, G, J) or TCA (B, E, H, K) and complete bile (C, F, I, J). GII.12A, black circle, GII.12A S392R magenta circle, GII.12A Q373E teal circle, GII.12B purple square, GII.12B R392S blue square, and GII.12B E373Q teal square. Error bars, standard error of the mean.

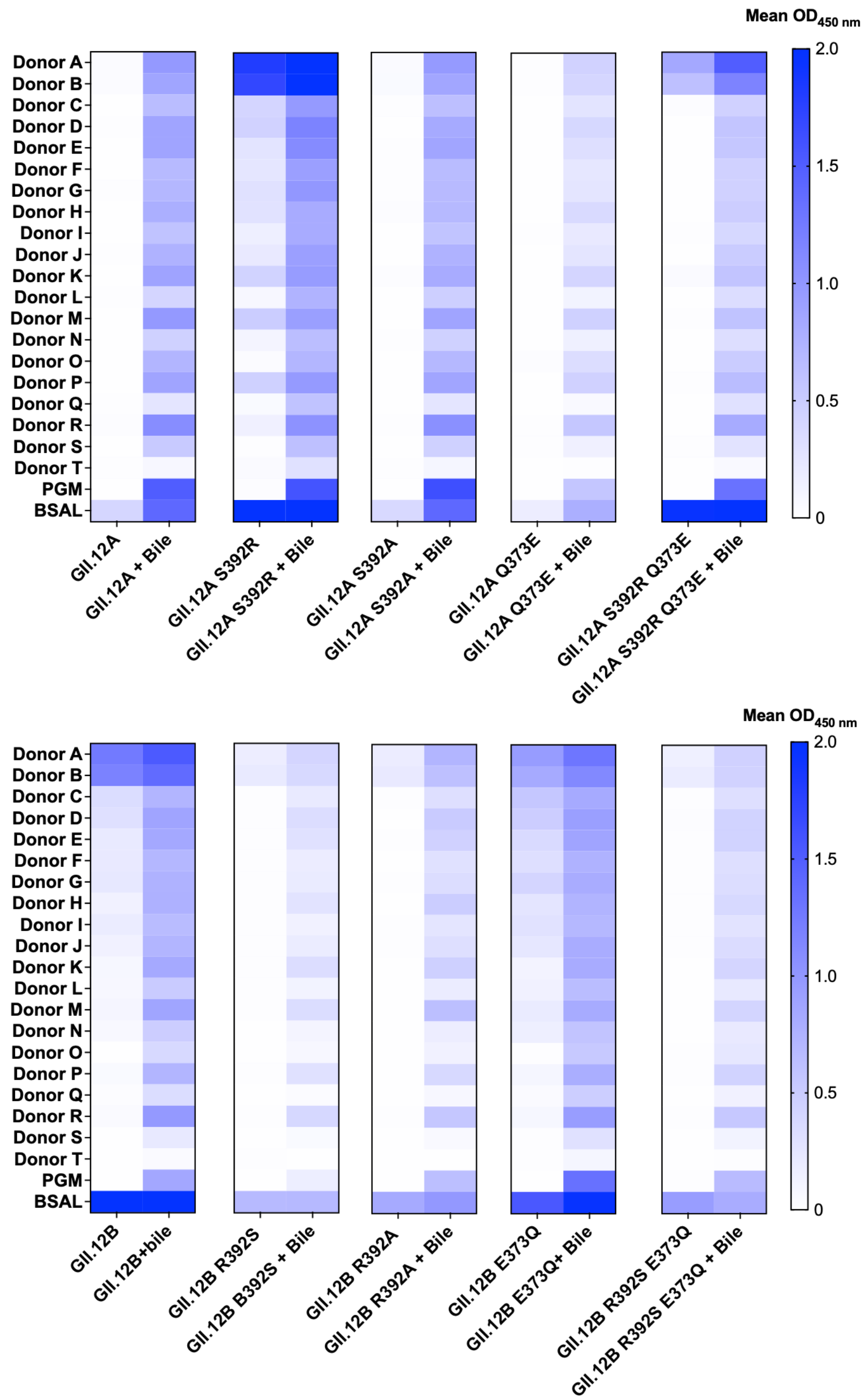

Figure S5: GII.12 Residues 392 and 373 influence binding to a panel of secretor positive human saliva: Saliva from gender unspecified, adult human donors were screened for alpha-1,2 fucose (secretor-positive) by reactivity to UEA-1-HRP. Saliva samples positive for UEA-1 binding, n=20, were tested for binding of wildtype GII.12A and GII.12B VLP, and VLP with mutations at either or 392 and 373 in the absence or presence of 1% bile. VLP binding visualized as heat maps with each rectangular bar representing the mean OD_{450nm} of four technical repeats. Donors organized by highest to lowest detectable binding, Blue to white, respectively.

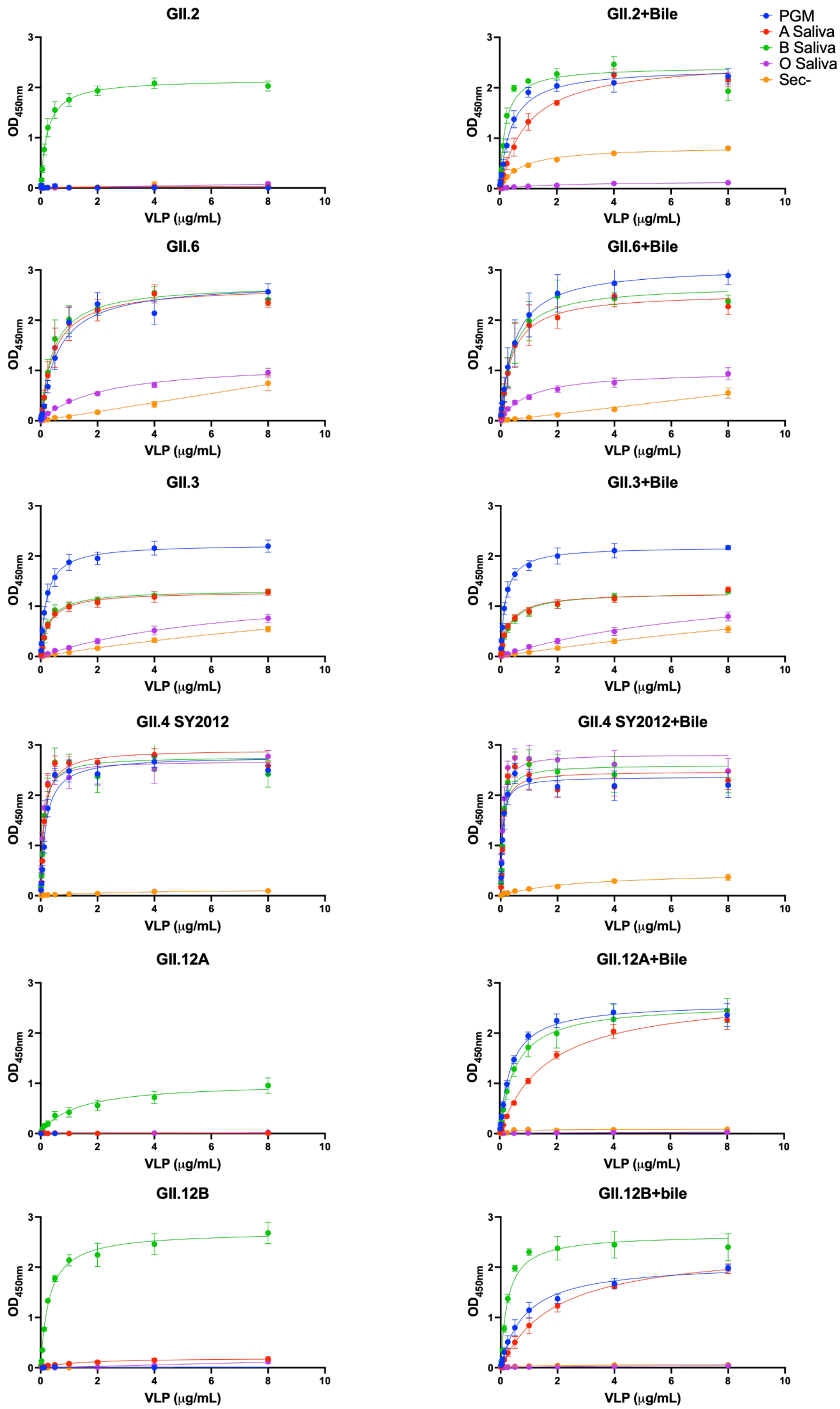
GII.2
GII.2+Bile
PGM
A Saliva
B Saliva
O Saliva
Sec-
GII.6
GII.6+Bile
GII.3
GII.3+Bile
GII.4 SY2012
GII.4 SY2012+Bile
GII.12A
GII.12A+Bile
GII.12B
GII.12B+bile
OD_{450nm}
VLP (μg/mL)
0
1
2
3
0
2
4
6
8
10

Figure S6: GII norovirus recognition of ligands in the presence of 1% bile. GII norovirus VLP were titrated against PGM or archival salivary samples of known HBGA expression profiles. PGM (blue), A, B, and O saliva (red, green, purple), secretor negative saliva (orange), with and without 1% bile. Data were analyzed using one-site specific non-linear regression curve fit analysis, markers representing mean OD_{450nm} with error bars representing standard error of the mean.

K_d µg/mL (SEM)

VLP	*PGM*	*PGM + Bile*	*B Saliva*	*B Saliva + Bile*
**GII.12A**	N/A	1.24 ± 0.10	1.24 ± 0.32	0.89 ± 0.08
**GII.12A Q373E**	N/A	1.89 ± 0.17	1.63 ± 0.31	2.31 ± 0.25
**GII.12A S392R**	N/A	1.59 ± 0.18	0.24 ± 0.02	0.20 ± 0.02
**GII.12A S392A**	N/A	1.58 ± 0.28	1.27 ± 0.25	0.94 ± 0.15
**GII.12A S392R Q373E**	N/A	2.07 ± 0.20	0.19 ± 0.02	0.16 ± 0.02
**GII.12B**	N/A	2.60 ± 0.19	0.30 ± 0.06	0.25 ± 0.04
**GII.12B E373Q**	N/A	1.64 ± 0.24	0.48 ± 0.02	0.25 ± 0.01
**GII.12B R392S**	N/A	1.41 ± 0.20	2.57 ± 0.35	2.51 ± 0.21
**GII.12B R392A**	N/A	2.68 ± 0.56	1.08 ± 0.09	1.02 ± 0.07
**GII.12B R392S E373Q**	N/A	1.67 ± 0.11	1.34 ± 0.20	2.74 ± 0.24

B_{max} OD_{450nm} (SEM)

VLP	*PGM*	*PGM + Bile*	*B Saliva*	*B Saliva + Bile*
**GII.12A**	N/A	2.42 ± 0.07	0.82 ± 0.07	2.31 ± 0.07
**GII.12A Q373E**	N/A	1.79 ± 0.06	0.87 ± 0.06	2.35 ± 0.10
**GII.12A S392R**	N/A	2.74 ± 0.11	2.73 ± 0.05	2.52 ± 0.04
**GII.12A S392A**	N/A	1.57 ± 0.10	0.50 ± 0.03	2.03 ± 0.10
**GII.12A S392R Q373E**	N/A	2.61 ± 0.10	2.82 ± 0.06	2.58 ± 0.08
**GII.12B**	N/A	1.36 ± 0.04	2.77 ± 0.06	2.29 ± 0.04
**GII.12B E373Q**	N/A	1.99 ± 0.11	2.57 ± 0.03	2.69 ± 0.04
**GII.12B R392S**	N/A	0.86 ± 0.04	2.42 ± 0.14	2.17 ± 0.08
**GII.12B R392A**	N/A	0.79 ± 0.07	2.59 ± 0.07	2.37 ± 0.05
**GII.12B R392S E373Q**	N/A	1.83 ± 0.04	2.29 ± 0.09	2.68 ± 0.09

Table S1: Calculated K_d and B_{max} for GII.12 VLP with standard margin of error. Standard margin of error (SEM) calculated from one-site specific binding analysis (Sup. Figure 3, Figure 2) of GII.12 VLP titration on PGM with or with without 1% bile for both K_d (µg/mL) and B_{max} (OD_{450nm}) values. N/A, VLP binding data not applicable.

PGM K_d μg/mL with Bile Comparisons

VLP	***Significance***	***p value***
**GII.12A vs GII.12B**	**	p=.0019
**GII.12A vs GII.12A Q373E**	*	p=.0451
**GII.12A vs GII.12A Q373E S392R**	**	p=.0080
**GII.12A vs GII.12A S392A**	NS	p=.3511
**GII.12A vs GII.12A S392R**	NS	p=.2222
**GII.12B vs GII.12B E373Q**	***	p=.0009
**GII.12B vs GII.12B E373Q R392S**	*	p=.0379
**GII.12B vs GII.12B R392A**	NS	p=.9551

PGM B_{max} OD_{450nm} with Bile Comparisons

VLP	***Significance***	***p value***
**GII.12A vs GII.12B**	***	p=.0002
**GII.12B vs GII.12B E373Q**	*	p=.0206
**GII.12B vs GII.12B E373Q R392S**	***	p=.0002
**GII.12B vs GII.12B R392S**	**	p=.0016
**GII.12B vs GII.12B R392A**	***	p=.0006
**GII.12A vs GII.12A Q373E**	**	p=.0062
**GII.12A vs GII.12A S392A**	**	p=.0025
**GII.12A vs GII.12A S392R**	NS	p=.1274
**GII.12A vs GII.12A Q373E S392R**	NS	p=.3450

B Saliva K_d μg/mL without Bile Comparisons

VLP	***Significance***	***p value***
**GII.12B vs GII.12A**	**	p=.0095
**GII.12B vs GII.12A Q373E**	**	p=.0095
**GII.12B vs GII.12B R392S**	*	p=.0159
**GII.12B vs GII.12B E373Q R392S**	**	p=.0095
**GII.12B vs GII.12A S392A**	**	p=.0015
**GII.12B vs GII.12B R392A**	**	p=.0061
**GII.12A vs GII.12A S392R**	**	p=.0043

**GII.12A vs GII.12A Q373E S392R**	**	p=.0022
**GII.12A vs GII.12B E373Q**	***	p=.0010
**GII.12B vs GII.12A S392R**	NS	p=.4127
**GII.12B vs GII.12A Q373E S392R**	NS	p=.1143
**GII.12B vs GII.12B E373Q**	NS	p=.0539

B Saliva B_{max} OD_{450nm} without Bile Comparisons

VLP	***Significance***	***p value***
**GII.12B vs GII.12A**	*	p=.0159

B Saliva K_d µg/mL with and without Bile Comparisons

VLP	***Significance***	***p value***
**GII.12A vs GII.12A with bile**	NS	p=.3543
**GII.12A S392A vs GII.12A S392A with bile**	NS	p>.9999
**GII.12A Q373E vs GII.12A Q373E with bile**	NS	p=.2468
**GII.12B vs GII.12B with bile**	NS	p=.4857
**GII.12B E373Q vs GII.12B E373Q with bile**	****	p<.0001
**GII.12B R392S vs GII.12B R392S with bile**	NS	p>.9999
**GII.12B R392A vs GII.12B R392A with bile**	NS	p=.8048

B Saliva B_{max} OD_{450nm} with and without Bile Comparisons

VLP	***Significance***	***p value***
**GII.12A vs GII.12A with bile**	**	p=.0016
**GII.12A Q373E vs GII.12A Q373E with bile**	**	p=.0043
**GII.12A S392A vs GII.12A S392A with bile**	****	p<.0010

Table S2: PGM and B Saliva K_d and B_{max} Statistical comparisons between GII.12 mutants. Calculated significance and p values for Figure 2 in-text K_d (µg/mL) and B_{max} (OD_{450nm}) comparisons obtained via Mann-Whitney text. Analyzed K_d and B_{max} values obtained from one-site specific non-linear regression curve fit analysis for each technical VLP-ligand titration run per VLP compared via Mann-Whitney test.

	Average VLP-Human Salivary Sample Binding OD_{450nm} (SEM)			
Parental Strain	***VLP Mutation Site***	***Without Bile***	***With Bile***	***Difference***
**GII.12A**	**-**	0.01 ± 0.00	0.70 ± 0.05	0.69
	**Q373E (Polar uncharged to Acidic)**	0.01 ± 0.00	0.29 ± 0.02	0.29
	**S392R (Polar uncharged to Basic)**	0.39 ± 0.06	0.97 ± 0.06	0.58
	**S392A (Polar uncharged to nonpolar)**	0.02 ± 0.00	0.68 ± 0.04	0.66
	**Q373E S392R**	0.08 ± 0.03	0.55 ± 0.04	0.47
**GII.12B**	**-**	0.23 ± 0.04	0.72 ± 0.04	0.48
	**E373Q (Acidic to polar uncharged)**	0.28 ± 0.03	0.74 ± 0.04	0.46
	**R392S (Basic to polar uncharged)**	0.03 ± 0.01	0.22 ± 0.02	0.19
	**R392A (Basic to nonpolar)**	0.03 ± 0.01	0.35 ± 0.03	0.32
	**E373Q R392S**	0.03 ± 0.01	0.33 ± 0.02	0.30

Table S3: OD_{450nm} binding signal differences for GII.12 VLP against human salivary samples. Reported average OD_{450nm}, margin of error, and difference in OD_{450nm} calculated for GII.12 mutant VLP binding to donor human saliva (sup. Figure 5) with or without 1% bile.

VLP	Without Bile K_d µg/mL (SEM)				
	PGM	A Saliva	B Saliva	O Saliva	Sec- Saliva
**GII.2**	N/A	N/A	0.23 ± 0.03	N/A	N/A
**GII.6**	0.61 ± 0.12	0.46 ± 0.10	0.43 ± 0.08	2.13 ± 0.31	N/A
**GII.3**	0.21 ± 0.03	0.30 ± 0.04	0.29 ± 0.04	7.06 ± 2.14	22.41 ± 11.28
**GII.4 SY2012**	0.18 ± 0.03	0.13 ± 0.02	0.10 ± 0.02	0.08 ± 0.01	N/A
**GII.12A**	N/A	N/A	1.24 ± 0.38	N/A	N/A
**GII.12B**	N/A	N/A	0.30 ± 0.04	N/A	N/A

VLP	With Bile K_d µg/mL (SEM)				
	PGM	A Saliva	B Saliva	O Saliva	Sec- Saliva
**GII.2**	0.39 ± 0.06	0.98 ± 0.14	0.20 ± 0.03	N/A	0.72 ± 0.06
**GII.6**	0.47 ± 0.13	0.40 ± 0.11	0.40 ± 0.11	0.99 ± 0.20	N/A
**GII.3**	0.17 ± 0.02	0.31 ± 0.04	0.35 ± 0.05	8.13 ± 2.62	25.33 ± 15.54
**GII.4 SY2012**	0.06 ± 0.02	0.07 ± 0.02	0.08 ± 0.02	0.07 ± 0.01	N/A
**GII.12A**	0.39 ± 0.05	1.65 ± 0.19	0.53 ± 0.10	N/A	N/A
**GII.12B**	0.85 ± 0.13	1.91 ± 0.28	0.25 ± 0.04	N/A	N/A

VLP	Without Bile B_{max} OD_{450nm} (SEM)				
	PGM	A Saliva	B Saliva	O Saliva	Sec- Saliva
**GII.2**	N/A	N/A	2.16 ± 0.07	N/A	N/A
**GII.6**	2.77 ± 0.16	2.68 ± 0.16	2.72 ± 0.14	1.17 ± 0.07	0.75 ± 0.36
**GII.3**	2.24 ± 0.07	1.29 ± 0.04	1.32 ± 0.04	1.43 ± 0.25	0.55 ± 0.11
**GII.4 SY2012**	2.77 ± 0.10	2.91 ± 0.09	2.77 ± 0.12	2.68 ± 0.09	N/A
**GII.12A**	N/A	N/A	1.02 ± 0.11	N/A	N/A
**GII.12B**	N/A	N/A	2.71 ± 0.09	N/A	N/A

VLP	With Bile B_{max} OD_{450nm} (SEM)				
	PGM	A Saliva	B Saliva	O Saliva	Sec- Saliva
**GII.2**	2.40 ± 0.09	2.57 ± 0.12	2.42 ± 0.09	N/A	0.83 ± 0.02
**GII.6**	3.08 ± 0.23	2.55 ± 0.18	2.70 ± 0.18	0.99 ± 0.06	0.55 ± 0.25
**GII.3**	2.19 ± 0.06	1.27 ± 0.04	1.28 ± 0.05	1.59 ± 0.31	0.55 ± 0.13
**GII.4 SY2012**	2.37 ± 0.11	2.48 ± 0.11	2.61 ± 0.12	2.82 ± 0.11	0.48 ± 0.05
**GII.12A**	2.60 ± 0.08	2.79 ± 0.12	2.59 ± 0.14	N/A	N/A
**GII.12B**	2.10 ± 0.10	2.42 ± 0.13	2.65 ± 0.10	N/A	N/A

Table S4: Calculated K_d and B_{max} for GII VLP with standard margin of error. Standard margin of error (SEM) calculated from one-site specific non-linear regression curve fit analysis

(Sup. Figure 6, Figure 4) of GII VLP titration on PGM with or with without 1% bile for both K_d and B_{max}. N/A, VLP binding data not applicable.
